# Transperineal intraprostatic injection of botulinum neurotoxin A vs transurethral resection of prostate for management of lower urinary tract symptoms secondary to benign prostate hyperplasia: A prospective randomised study

**DOI:** 10.1080/2090598X.2019.1662214

**Published:** 2019-10-03

**Authors:** Amr S. El-Dakhakhny, Tarek Gharib, Ahmed Issam, Tarek M. El-Karamany

**Affiliations:** a Department of Urology, Faculty of Medicine, Benha University, Benha, Egypt; b Department of Urology, Faculty of Medicine, Minia University, Minia, Egypt

**Keywords:** Botulinum toxin A, intraprostatic injection, TURP, outcome, urodynamic studies, erectile function

## Abstract

**Objectives**: To evaluate transperineal intraprostatic injection of botulinum neurotoxin A (BoNT-A) in patients with lower urinary tract symptoms (LUTS) secondary to benign prostate hyperplasia (BPH) who failed to respond to 6-month medical treatment compared with transurethral resection of the prostate (TURP).

**Patients and methods**: In all, 92 men were divided into TURP and BoNT-A groups after being evaluated using the International Prostate Symptom Score (IPSS) and five-item version of the International Index of Erectile Function, estimation of serum total prostate-specific antigen (tPSA), ultrasonographic estimation of prostatic volume (PV), and uroflowmetry determination of voiding volume (VV), maximum urinary flow rate (Q_max_) and post-void residual urine volume (PVR). BoNT-A (200 U diluted in 3 mL saline) was injected, using a 22-G spinal needle under transrectal ultrasonography guidance, with 1-mL in each lobe. Patients were assessed 3-monthly for 12 months.

**Results**: The IPSS significantly decreased in all patients with a non-significant difference between the groups. The mean VV and Q_max_ increased, whilst PVR, PV and serum tPSA significantly decreased. Patients who showed deterioration at 12 months were re-evaluated and underwent TURP. BoNT-A injection significantly maintained erectile function compared with TURP.

**Conclusion**: Intraprostatic BoNT-A injection reduced PV with subsequent increases in VV and Q_max_, and decreases in PVR and serum tPSA level. Intraprostatic BoNT-A injection allowed surgery sparing in >70% and preserved erectile function in 91.3% of patients.

**Abbreviations:** BoNT-A: botulinum neurotoxin A; HRQOL: health-related quality of life; IIEF-5: five-item version of the International Index of Erectile Function; PV: prostatic volume; PVR: post-void residual urine volume; Q_max_: maximum urinary flow rate; tPSA: total PSA; VV: voided volume

## Introduction

Bladder outlet anatomical structures in men include the bladder neck, urethral sphincter and prostate [1]. BOO results in slow urinary flow and increased intravesical pressure with concomitant back pressure [2]. BPH is a prevalent disease amongst ageing males [3] and its urinary complications continue to pose serious health problems [4], which deleteriously affect ageing men’s health-related quality of life (HRQOL) [5].

Surgical management of BPH is indicated in medical non-responders, presenting with advanced signs of BOO and obstructive uropathy [6]. Simple open prostatectomy was the traditional management option [7] for improving LUTS, but at the expense of considerable surgical and perioperative morbidity [8].

Minimally invasive management of male LUTS due to BOO aim to provide equal effectiveness as standard techniques with a more favourable safety profile [9]. TURP is the ‘gold standard’ method for surgical treatment of BPH [10], but other procedures such as photoselective prostate vaporisation [11] and bipolar transurethral enucleation are effective surgical options [12].

*Clostridium botulinum* produces seven, A–G, immunologically distinct neurotoxins [13], but botulinum neurotoxin A (BoNT-A) is the most biologically potent and is the most commonly used [14]. BoNT-A is a double-chain; light and heavy, protein connected by a disulphide bond [15]. In the presynaptic nerve membrane, the C-terminal of the heavy chain binds to synaptic vesicle protein 2 and the toxin is taken into the nerve terminal by endocytosis [16]. The light chain inhibits acetylcholine release by disrupting the fusion of vesicles with the neurone cell membrane, finally causing the flaccid paralysis of muscles [17]. For treatment of LUTS, BoNT-A significantly improves all symptoms and urodynamic parameters in neurogenic detrusor overactivity and overactive bladder [18], and offers an effective treatment option for patients with refractory overactive bladder [19]. In the present study, we evaluated the subjective and objective outcomes of transperineal intraprostatic BoNT-A injection, in patients with LUTS secondary to BPH who failed to respond to medical treatment, in comparison to TURP.

## Design

Prospective comparative clinical trial.

## Setting

University Hospital.

## Patients and methods

The protocol of the present study was approved by the Local Ethics Committee and all enrolled patients signed written fully informed consent before study inclusion. Men aged >50 years who presented to the Urology Outpatient Clinic with LUTS secondary to BPH and failed to respond to conservative therapy for 6 months, were eligible for evaluation.

All patients were evaluated for demographic and clinical data, and were evaluated subjectively using the IPSS, HRQOL due to urinary symptoms [20], and the five-item version of the International Index of Erectile Function (IIEF-5), for assessment of erectile function [21]. All patients gave a fasting blood sample, before any prostatic manipulations, for estimation of serum total PSA (tPSA), and after assurance of diagnosis of BPH, all patients underwent TRUS for evaluation of total prostatic volume (PV), volume of prostatic adenoma, and exclusion of the presence of prostate cancer or prostatitis. Thereafter, patients underwent uroflowmetry determination of voided volume (VV), maximum urinary flow rate (Q_max_) and post-void residual urine volume (PVR). Lastly, all patients underwent cystoscopic bladder examination and bladder biopsy was obtained for histopathological examination to exclude malignancy.

Inclusion criteria included prostatic enlargement up to or >30 mL in volume, IPSS of ≥8, serum tPSA of <10 ng/mL, Q_max_ of ≤15 mL/s with a VV of ≥150 mL, and cystoscopic bladder biopsy was negative for malignancy. Exclusion criteria included previous prostate ablative treatment, neurogenic voiding disorder, urethral stricture, prostatitis, chronic bladder catheterisation, contraindication for BoNT-A administration, or PVR of >250 mL. Enrolled patients were randomly, using sealed envelopes prepared by a blinded assistant and chosen by the patient, divided into two equal groups: TURP group, included patients undergoing TURP and the BoNT-A group included patients receiving transperineal intraprostatic BoNT-A injection

### Procedure of transperineal intraprostatic BoNT-A injection

Injection fluid preparation, 200 U BoNT-A (OnaBotA; Botox Allergan, Dublin, Ireland) was diluted in 3 mL saline. All procedures were conducted under i.v. light sedation; broad-spectrum antibiotic was given i.v. as prophylaxis. The patient was positioned in lithotomy and the perineal region was sterilised with povidone iodine, and using TRUS guidance a 22-G spinal needle was inserted transperineally and 1-mL of the prepared solution was injected in each lobe [22]. Patients were asked to continue drug therapies for BPH for 3-weeks after which the clinical effect of the BoNT-A injection should manifest, as documented by Marchal et al. [23].

### Study outcomes

- Primary outcome included subjective improvement as evaluated by IPSS at 3, 6, 9 and 12 months after intervention in comparison to baseline IPSS for each group and between both groups.
- Secondary outcomes included changes in:
  1. VV, Q_max_, PVR, serum tPSA levels and PV at 3, 6, 9 and 12 months after injection.
  2. Changes in IIEF-5 score at the 12-month follow-up in relation to the baseline score.

### Sample size calculation

Previous studies [24,25] compared IPSS at 12 months after BoNT-A injection to baseline scores and reported improved IPSS by 49% [24] and 45% [25]. The sample size was calculated using G*Power software, version 3.1.9.4 (University of Düsseldorf, Düsseldorf, Germany) and with test family (*t*-tests) statistical test (difference between two independent means for two groups), type of power analysis (a priori: compute required sample size, given α, power, and effect size). Based on the hypothesis that the IPSS is expected to be improved by 45% in Group 1 and 50% in Group 2, the effect size determined by the G*Power software was 0.55. With α error of 0.05, and power (1–β) of 0.8, and allocation ratio N2/N1 of 1, the sample size for each group was 53 cases. Given an expected drop out of 10%, the total sample size was expected at 118; 59 cases in each arm.

### Statistical analysis

The data are presented as mean (SD), numbers and percentages. Results were analysed using the one-way ANOVA, Student’s *t*-test and chi-squared test. Statistical analysis was conducted using the IBM Statistical Package for the Social Sciences (SPSS®; version 23, 2015) for Windows (SPSS Inc., IBM Corp., Armonk, NY, USA). A *P* < 0.05 was considered statistically significant.

## Results

**Figure 1.**
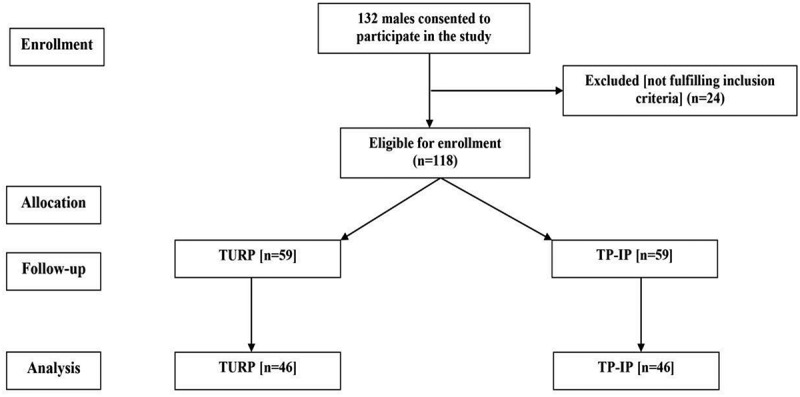
Consolidated Standards of Reporting Trials (CONSORT) diagram. TP-IP, transperineal intraprostatic BoNT-A injection.

The study included 92 patients fulfilling the inclusion criteria (Figure 1). Patients’ enrolment data showed non-significant (*P* > 0.05) differences between both groups (Table 1).

**Table 1.** Patients’ enrolment data.

Variable	TURP group	Intraprostatic BoNT-A group	*P*
Number of patients	46	46	
Mean (SD):			
Age, years	61.3 (6.1)	59 (5.5)	0.061
Body mass index (BMI) data			
Weight, kg	87.5 (7)	89.1 (8.1)	0.139
Height, cm	171.5 (2.9)	171.8 (3.2)	0.782
BMI, kg/m^2^	29.8 (2.3)	30.2 (2.6)	0.236
Subjective evaluation			
IPSS	21.5 (3.7)	20.2 (4.2)	0.101
HRQOL score	4 (1.4)	3.7 (1.7)	0.385
IIEF-5 score	17.2 (4.9)	16.1 (4.4)	0.236
Objective measures			
VV, mL	253.2 (88.9)	261.2 (83.9)	0.674
PVR, mL	77.2 (35.2)	73.9 (34)	0.417
Q_max_, mL/s	8.1 (2.9)	9.3 (3.5)	0.077
PV, mL	42.5 (13)	45.2 (16.8)	0.343
TRUS data			
PV, mL	50.5 (19.7)	45.2 (16.8)	0.277
Adenoma volume, mL	28.2 (13.1)	31.2 (10)	0.221
tPSA, mg/mL	3.1 (2)	3.2 (2.1)	0.918

**Figure 2.**
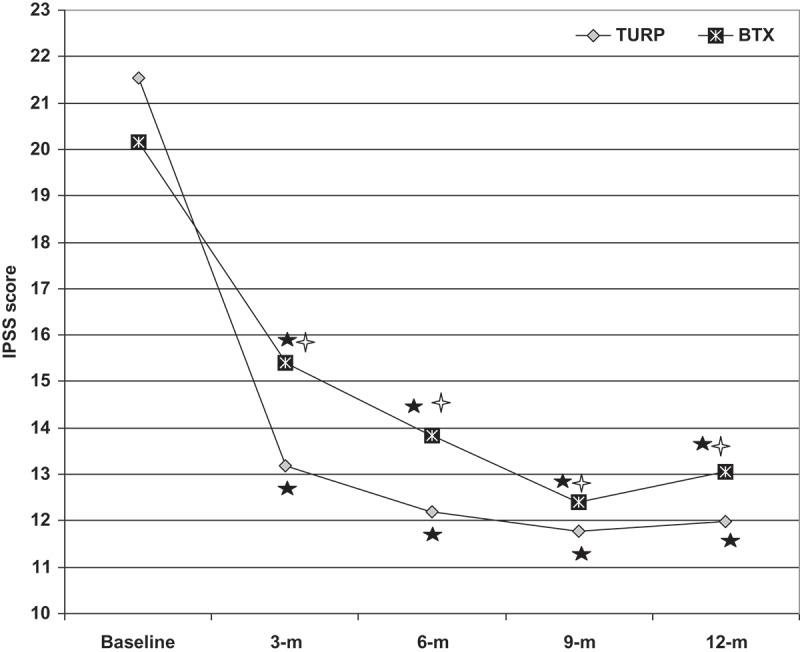
The mean IPSS of patients of both groups determined during follow-up compared to preoperative score (black star, indicates significant difference vs preoperative score); open cross, indicates significant difference vs score of TURP patients. m, months. BTX, BoNT-A.

Considering the primary outcome as the improvement in the IPSS, throughout the 12-month follow-up all patients showed progressive subjective improvement as evidenced by the significantly lower IPSS in comparison to baseline scores. However, improvement progressed more slowly with BoNT-A injection, as shown by the significantly higher IPSS of patients who received injections at the 3 and 6-month follow-up visits, but the difference became non-significant at the 9- and 12-month follow-ups in comparison to patients who received TURP. In both groups of patients, the extent of improved IPSS peaked at the 9-month follow-up, with a significantly higher percentage score improvement with TURP than with BoNT-A injection (Table 2, Figure 2).

**Table 2.** IPSS data and patients’ comments on prostate-related manifestations throughout the 12-month follow-up.

Variable	TURP group (*n* = 46)	Intraprostatic BoNT-A group (*n* = 46)	*P*
Mean (SD):			
Baseline IPSS	21.5 (3.7)	20.2 (4.2)	
IPSS at 3 months	13.2 (2.7)*	15.4 (3.8)*	0.002
% improvement	38.5 (8.9)	23.1 (12.2)	0.001
IPSS at 6 months	12.2 (2.7)*	13.8 (3.4)*	0.012
% of improvement	43.5 (6.8)	30.9 (11.4)	0.003
IPSS at 9months	11.8 (2.1)*	12.4 (3.2)*	0.269
% of improvement	44.2 (10.9)	37.5 (13.1)	0.029
IPSS at 12 months	12 (2.5)*	13.1 (3.9)*	0.115
% of improvement	43.3 (12.3)	34.1 (17)	0.007
Progress of prostate-related manifestations, *n* (%)			
At 3 months			1
Improved	46 (100)	46 (100)	
Static	0	0	
Deteriorated	0	0	
At 6 months			<0.001
Improved	0	31 (67.4)	
Static	43 (93.5)	15 (32.6)	
Deteriorated	3 (6.5)	0	
At 9 months			0.001
Improved	0	25 (54.3)	
Static	40 (87)	13 (28.3)	
Deteriorated	6 (13)	8 (17.4)	
At 12 months			0.027
Improved	0	8 (17.4)	
Static	38 (82.6)	28 (60.9)	
Deteriorated	8 (17.4)	10 (21.7)	

*Significant difference vs baseline estimates.

At the 12-month follow-up; eight patients in the BoNT-A group were still showing improvement, 13 patients were static since the 6-month scoring, and 15 patients were static since the 9-month scoring, whilst 10 patients had deteriorated IPSS. On the other hand, 38 patients in TURP group had static IPSS, while eight had a deteriorated score. Patients of both groups showed significant differences for the extent of change in the IPSS at end of follow-up, which was in favour of the BoNT-A group (Table 2).

**Figure 3.**
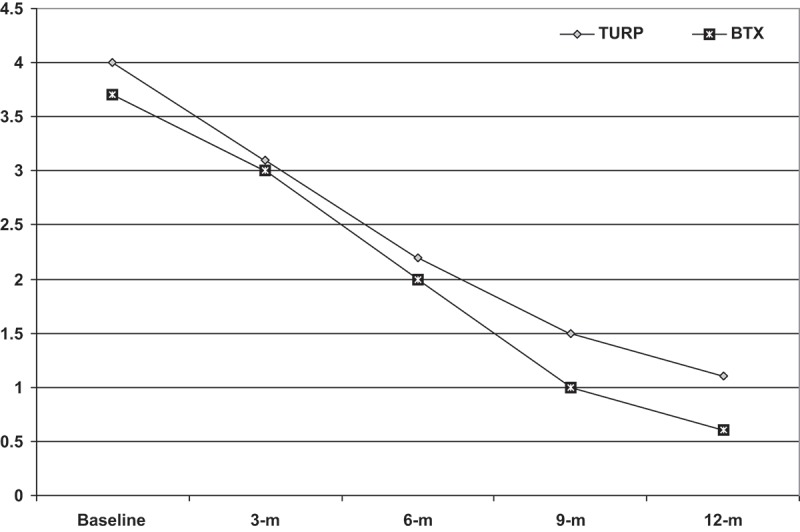
The mean HRQOL scores of both groups. m, months. BTX, BoNT-A.

**Figure 4.**
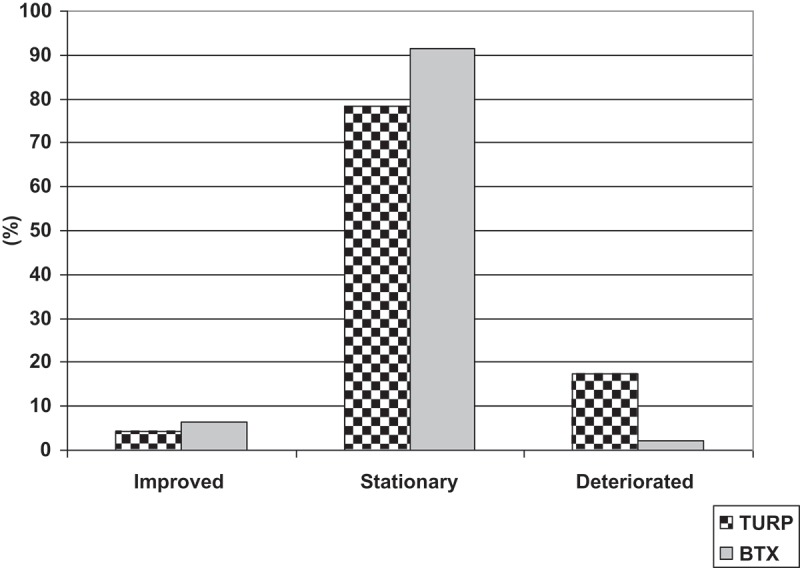
Patients’ distribution according to effect of treatment on erectile function. BTX, BoNT-A.

The HRQOL score progressively decreased in all patients with non-significant difference between both groups at the 3-month follow-up, but thereafter the difference became significant in favour of the BoNT-A injection procedure. Moreover, at the 12-month follow-up, 38 patients had a score of zero; 14 and 24 in TURP and BoNT-A groups, respectively, with a significant difference in favour of BoNT-A group (Figure 3). On contrary, the IIEF-5 scores were not significantly higher in patients in both groups compared to their baseline scores, with a non-significant difference in favour of the BoNT-A group. Amongst patients who received BoNT-A injection therapy, 42 had preserved erectile function, three had improved, and only one had deteriorated erectile function. Conversely, amongst patients who had TURP, two patients had improved, eight had deteriorated, while 36 had preserved erectile function with a significant difference in favour of the BoNT-A group (*P* = 0.047; Table 3, Figure 4).

**Table 3.** Urinary HRQOL and IIEF-5 data of studied patients.

Variable	TURP group (*n* = 46)	Intraprostatic BoNT-A group (*n* = 46)	*P*
HRQOL score			
Baseline score, mean (SD)	4 (1.4)	3.7 (1.7)	0.385
Score at 3 months			
mean (SD)	3.1 (1.1)	3 (1.2)	0.478
% of improvement	18.3 (18.9)	16 (15.5)	0.521
Score at 6 months			
mean (SD)	2.2 (1.4)	2 (0.9)	0.011
% of improvement	47.8 (24.8)	49.7 (13)	0.643
Score at 9 months			
mean (SD)	1.5 (1)	1 (0.9)	0.011
% of improvement	63 (22.5)	77.5 (21)	0.001
Score at 12 months			
mean (SD)	1.1 (0.9)	0.6 (0.7)	0.003
% of improvement	71.8 (23)	86.3 (16)	<0.001
Number of patients with a score of zero at 12 months (%)	14 (30.4)	24 (52.2)	0.034
IIEF-5			
Mean (SD)			
Baseline IIEF-5	17.2 (4.9)	16.1 (4.4)	0.236
IIEF-5 at 12 months	18.2 (5)	18.4 (5)	0.567
Change in erectile function, *n* (%)			0.047
Improved	2 (4.3)	3 (6.4)	
Static	36 (78.3)	42 (91.4)	
Deteriorated	8 (17.4)	1 (2.2)

**Figure 5.**
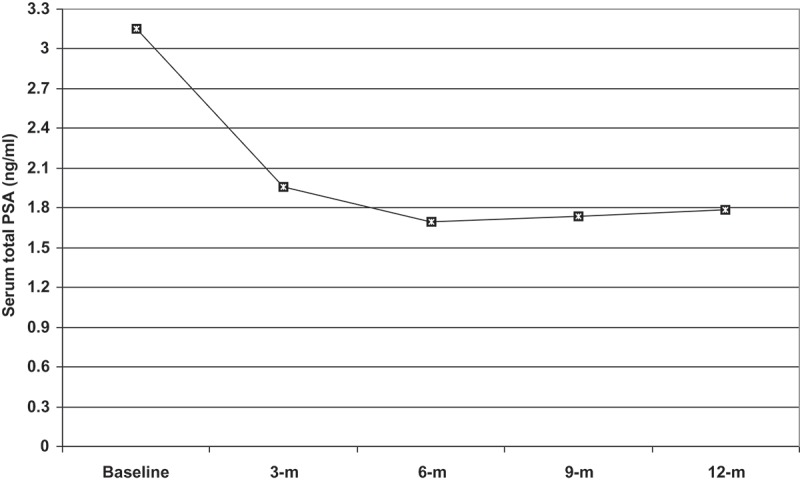
The mean serum tPSA levels in the patients who received intraprostatic BoNT-A injections throughout the 12-month follow-up. m, months.

For the BoNT-A group, the mean VV progressively increased and PVR decreased throughout the 12-month follow-up, with significant differences vs baseline volumes. The volume change peaked at the 6-month follow-up, with a significant difference vs all other estimates. The mean Q_max_ at the 9- and 12-month follow-ups was significantly higher compared to all previous estimates; and at the 3- and 6-month follow-ups vs baseline estimates, with non-significantly higher estimates at 6 months than at 3 months. The estimated PV showed a gradual decrease with a significant difference compared to baseline PV and reached its smallest volume at the 12-month follow-up, which was significantly smaller than all previous estimates (Table 4). The mean serum tPSA levels decreased significantly at all follow-up times compared with the baseline level and reached their lowest level at the 6-month follow-up, with significantly (*P* = 0.044) lower levels (mean [SD] 1.7 [0.51] ng/mL) compared to the 3-month follow-up levels (mean [SD] 1.96 [0.56] ng/mL), and changed non-significantly thereafter (Figure 5).

**Table 4.** Follow-up urodynamic and TRUS data of patients in the Intraprostatic BoNT-A group compared to baseline data.

		Follow-up at:			
Variable, mean (SD)	Baseline	3 months	6 months	9 months	12 months
VV, mL	261.2 (83.9)	354.7 (131.2)*	411.9 (123.6)*†	367.9 (129.3)*	364.2 (141.6)*
PVR, mL	73.9 (34)	40.9 (24.5)*	22.6 (5.2)*†	24.7 (10.3)*†	25.8 (9.3)*†‡
Q_max_, mL/s	9.3 (3.5)	13 (2.8)*	13.8 (3)*	14.4 (2.9)*†‡	16.6 (2.2)*†‡ʃ
PV, mL	45.2 (16.8)	37 (11.3)*	34.1 (9)*	29 (8.1)*†‡	27.3 (5.9)*†‡ʃ

*Significant difference vs baseline estimates; †significant difference vs 3-month estimates; ‡significant difference vs 6-month estimates; ʃsignificant difference vs 9-month estimates.

Patients who had a deterioration in IPSS at the 12-month follow-up were re-evaluated for urodynamic parameters and by TRUS, and 10 patients in the BoNT-A group were prepared and underwent TURP, thus, transperineal injection of BoNT-A spared surgical intervention in 36 patients (78.3%), who were satisfied with their outcome and accepted to continue follow-up; and postponed surgery in the 10 patients who were unsatisfied by their BoNT-A injection outcome. Conversely, eight patients in the TURP group had a secondary look and five required frequent dilatation, and three had re-resection for recurrent growth of the prostatic adenoma.

## Discussion

TURP as previously documented became the ‘gold standard’ surgical treatment for BPH [10,11,26–28], thus the present study evaluated the outcome of patients with LUTS secondary to BPH (LUTS/BPH) using transperineal intraprostatic BoNT-A injection in comparison to TURP.

Patients in the BoNT-A group received 200 U BoNT-A, the choice of the injection dose was dependent on previous findings; Crawford et al. [29] found that BoNT-A injection of 100 or 300 U BoNT-A is effective and safe, but the 100 U dose may be preferable due to similar efficacy with reduced costs and adverse effects. While, Arnouk et al. [30] detected a time course-dose dependent effect of BoNT-A injection of 100 and 200 U BoNT-A, as both doses produced significant subjective and objective improvements, but PV did not change significantly with 100 U, but was significantly reduced at the 6-month evaluation with a dose of 200 U. Moreover, multiple recent studies have reported the efficacy and safety of 200 U BoNT-A injection [31–33].

Concerning outcomes, BoNT-A injection did favourably in comparison to TURP, as shown by the non-significant differences in subjective scores determined throughout follow-up between patients of both groups. Moreover, the mean VV and Q_max_ progressively increased, whilst PVR, PV and serum tPSA progressively decreased throughout the 12-month follow-up, with significant differences vs baseline estimates.

The present results support those of early studies by Chartier-Kastler et al. [34], who reported that intraprostatic BoNT-A injections affect static and dynamic component of LUTS/BPH; and Marchal et al. [23] and Hamidi et al. [25] who reported significant decreases of PV, PVR and serum tPSA levels after intraprostatic injection of BoNT-A than before treatment.

The objective changes reported in patients who received BoNT-A injections had peaked at 6 months and then did not changed significantly in the majority of patients in the BoNT-A group. Similarly, Arnouk et al. [30] detected a significant difference in serum PSA levels at 6 months after intraprostatic 100 U BoNT-A injection, and after 3 and 6 months after injection of BoNT-A 200  U. Also, Ding et al. [31] detected remarkable improvement in LUTS/BPH at 1 month after injection, which reached optimal levels at 6 months and was maintained for ≥1 year

Patients who had deterioration in IPSS at the 12-month follow-up were re-evaluated for urodynamic parameters and using TRUS, and 10 patients in the BoNT-A group underwent TURP, thus, intraprostatic BoNT-A injection spared surgical intervention in 36 patients (78.3%) who were satisfied with their outcome and accepted to continue follow-up, and postponed surgery in the 10 patients who were unsatisfied by their BoNT-A injection outcome. Consistent with these figures, Rodrigues de Carvalho et al. [32] reported that intraprostatic BoNT-A injection could be an option for treating LUTS/BPH refractory to medical treatment in poor surgical candidates, preventing surgery in ~70% of patients with limited side-effects. Also, Andersson [35] considered intraprostatic botulinum toxin as an attractive minimally invasive surgical therapy for LUTS/BPH, which may have a potential as an alternative treatment to surgical procedures.

Recently, Totaro et al. [33] evaluated the effectiveness of BoNT-A in the treatment of patients with BPH who failed to respond to medical therapy and reported that subjective improvement started 1 month after injection and at the end of follow-up ~90% of patients reported subjective symptomatic relief and treatment satisfaction with no local or systemic side-effects.

Regarding erectile function as judged by the IIEF-5, BoNT-A injection resulted in superior outcomes manifested as a higher percentage of patients documented as having static or improved erectile function, with a significant difference vs the TURP patients (*P* = 0.047). These present findings go in hand with Magistro et al. [5] who searched randomised clinical trials evaluating outcome of botulinum toxin injection for the management of LUTS and concluded that it is a novel minimally invasive treatment with efficacy comparable to standard surgical techniques, often associated with more favourable safety profile, especially preservation of sexual function.

The PV reducing effect of BoNT-A injection with subsequent symptom relief could be explained by the mechanism of action of botulinum toxin in relation to prostatic physiological anatomy, where cholinergic nerves and muscarinic receptors that are expressed in prostatic fibromuscular stroma, have a role in prostatic tissue growth [36], so that blocked acetylcholine release from cholinergic nerves by botulinum [17] may lead to disrupted neural control of the prostate, inhibiting prostatic contraction and growth, and thus inducing symptomatic relief in men with BPH [35]. In line with this explanation, Smith et al. [37] reported that injected botulinum toxin inhibits urethral norepinephrine release and causes prostatic atrophy through selective denervation. Also, Oeconomou et al. [38] searched the literature regarding intraprostatic botulinum injection and found experimental studies reported that botulinum injection induced relaxation of the prostate, atrophy, and reduction of its size by inhibiting the trophic effect of the autonomic system on the prostate gland.

Experimentally, Hsu et al. [22] found that BoNT-A induces prostate apoptosis, down-regulation of α_1_ a1-adrenergic receptors, and reduces contractile function of the prostate. Also, Ergün et al. [39], using a rat model of BPH, detected decreases of estimated PV and its actual weight by ~32.5% after BoNT-A injection and attributed this to induction of prostate apoptosis.

## Conclusion

Transperineal intraprostatic BoNT-A injection improved LUTS/BPH by reducing PV with subsequent increases in VV and Q_max_ and a decrease in PVR. This beneficial effect peaks at 3–6 months after injection with a significant drop in serum tPSA. Intraprostatic BoNT-A injection therapy allowed surgery sparing in >70% of the patients with BPH and preserved erectile function in 91.3% of these patients. Thus, intraprostatic BoNT-A injection therapy could be a satisfactory option for patients unfit for surgery and young patients with acceptable erectile function, and patients refusing surgery.
